# Extracellular matrix alterations in experimental Leishmania amazonensis infection in susceptible and resistant mice

**DOI:** 10.1186/1297-9716-43-10

**Published:** 2012-02-08

**Authors:** Mariana Silva-Almeida, Luiz OP Carvalho, Ana L Abreu-Silva, Celeste SF Souza, Daiana J Hardoim, Kátia S Calabrese

**Affiliations:** 1 Laboratório de Imunomodulação e Protozoologia do Instituto Oswaldo Cruz/FIOCRUZ, Pavilhão 108, Av. Brasil, 4365, Manguinhos, CEP 21040-900, Rio de Janeiro, Brazil; 2 Departamento de Patologia da Universidade Estadual do Maranhão, São Luís, Maranhão, Brazil

## Abstract

*Leishmania *is inoculated, by the bite of an infected sandfly, into the skin of the host, where the promastigotes are phagocyted by dermal macrophages. The dermal region comprises cells and abundant extracellular matrix. Studies show that matrix metalloproteinases play an important role in host defense responses against pathogens in mammals and that their activities lead to the production of antimicrobial peptides. The aim of this study is to evaluate the changes in the distribution of fibronectin and laminin as well as in the elastic system fibres during the course of infection caused by *Leishmania amazonensis *in mice with distinct genetic backgrounds of susceptibility to this parasite. The results showed that BALB/c presented an enhancement of fibronectin during the course of infection when compared to their control group while the infected or non-infected C3H.He showed a decrease of this protein at end of the experiment. Laminin, on the other hand, remained unaltered in both strains. Also in both BALB/c and C3H.He mice the elastic and elaunin fibres remained unchanged while the oxytalan fibres decreased along the experiment. Ninety days after the infection C3H.He mice had recovered their capacity to produce oxytalan fibres.

## Introduction

Leishmaniasis represents a complex of diseases with important clinical and epidemiological diversity. Clinical forms of leishmaniasis are particularly diverse: visceral leishmaniasis (VL) is usually fatal when untreated, mucosal leishmaniasis (ML) is a mutilating disease, diffuse cutaneous leishmaniasis (DCL) is a long-lasting disease due to a deficient cellular-mediated immune response and cutaneous leishmaniasis (CL) is disabling when lesions are multiple [1]. Infected sandflies deposit *Leishmania *promastigotes within the host skin, after that the parasite is taken up into a membrane bound phagosome that is contiguous with the outer plasma membrane of the macrophage; hence, the parasite is initially in the extracellular environment [2].

The extracellular matrix is composed of a wide variety of different macromolecules which carry distinct domains with defined structural and/or biological activities. Cell-matrix interactions, therefore, not only control the shape and orientation of cells but can also directly or indirectly regulate cellular functions, including migration, differentiation, proliferation and the expression of different genes [3].

A copious elastic-fibre network is responsible for skin elasticity. These fibres extend from the dermal-epidermal junction as cascades of microfibril bundles (oxytalan fibres), through perpendicular elaunin fibres in the papillary dermis that contains small amounts of elastin, to thick horizontally aligned elastic fibres in the reticular dermis [4]. Collagen fibres, which are formed by a superfamily of 27 members divided into subgroups, provide tensile strength to the skin. At least three characteristics determine a collagen: the localization, always extracellular; the presence of at least one triple helix in its structure; and the ability to form macromolecular aggregates. These fibres are secreted by different cells, mostly from connective tissue, and represent 25% of the whole-body protein content of mammals [5].

Studies have shown that *Leishmania donovani *promastigotes express on their plasma membrane, molecules, like surface metalloprotease gp63, capable of recognizing the extracellular matrix (ECM) macromolecules which help them to glide through the interstitial tissue during their transit from blood to target cells [6,7] and *Leishmania mexicana *promastigotes have the ability of binding with type I collagen fibrils [8]. According to Bandyopadhyay et al. [9], the ability of the amastigotes to adhere to ECM components could be pivotal in the pathogenesis of visceral leishmaniasis. Otherwise, the metalloproteinases presented in dermal layer matrix play an important role in host defense responses against pathogens in mammals and their activities lead to the production of antimicrobial peptides. After dribbling the phagocytic mononuclear cells, the parasites need to surpass the ECM components. Thus, the ability of *Leishmania *to interact with ECM facilitates leishmaniasis pathogenesis.

The aim of this work is to analyze the changes in the distribution of ECM components as collagenous fibres, fibronectin and laminin as well as the elastic system fibres during *L. amazonensis *infection in mice with distinct genetic backgrounds of susceptibility to this parasite.

## Materials and methods

### Animals

Female BALB/c and C3H.He mice with ages ranging from 4 to 6 weeks old from the animal facilities of Oswaldo Cruz Foundation were used. There were twelve animals per experimental group. During the experiments, all mice were maintained under controlled temperature, receiving food and water *ad libitum*.

### Parasites

The H21 MHOM/BR/76/MA-76 strain of *L. amazonensis*, was isolated from a patient with diffuse cutaneous leishmaniasis and maintained by serial passages in mice in our laboratory: amastigotes were removed from footpad lesions, purified by filtering and inoculated subcutaneously (10^4 ^parasites/0.05 mL) in the footpads of mice.

### Experimental design

All experiments with animal were conducted in accordance with the guidelines for experimental procedures of Oswaldo Cruz Foundation (Licence no L.0001/07).

Mice were subcutaneously infected by injecting 10^4 ^*L. amazonensis *amastigotes in the left footpad. Lesion progression was recorded in five mice of each group by measuring footpad swelling (dorsal to plantar axis) with a vernier caliper. Lesion size was calculated by subtracting the thickness of uninfected contra-lateral hind footpad from the infected one. Lesion size of 5 mice was monitored, at 30, 60, 90, and 120 days post-infection (dpi) with a digital caliper (Schnelltaster, H.C. Kröplin, GMBH, Hessen, Germany). Three animals of each group were killed at 30, 60, 90 and 120 dpi. Mock-infected mice were used as controls. The results of three independent experiments showed no significant differences.

### Histopathology

The footpad lesion of each animal was removed and divided in two fragments. One fragment was washed in PBS and fixed in paraformaldehyde 4% in PBS 0.01 M, pH 7.45, 4°C for 48 h. Tissues fragments were processed for paraffin embedding. Five micrometers thick sections were stained with Haematoxylin-Eosin (H&E), or Masson's Trichrome, or picrosirius red (Direct Red 80, Aldrich Milwaukee, WI 53233, USA), or Gomori's method or Weigert's resorcin-fuchsin after monopersulphate compound (Sigma-Aldrich, Germany) oxidation [10] counterstained with Orange G.

### Immunohistochemistry

Fragments of footpad at 30 and 120 dpi were used for immunohistochemistry. Tissues were immediately embedded in OCT (OCT Compound-embedding medium for frozen specimens, Milles INC., USA) and frozen at -70°C for confocal laser scanning microscopy. Five micrometers thick cryosections were blocked and fixed in cold acetone for 15 min. The sections were blocked in phosphate saline buffer (PBS) containing 0.2% gelatine, 0.1% NaN_3_, and 0.1% saponin (PGN-saponin) to avoid nonspecific interactions, incubated with human polyclonal anti-*Leishmania *serum diluted 1:500, washed three times with PBS and then incubated with secondary Cy3-conjugated antibodies anti-human IgG (C2571 - Sigma; diluted 1:100), then the material was washed again. The slides were subsequently incubated with monoclonal antibodies against the ECM proteins: rabbit anti-fibronectin (F3648 - Sigma; diluted 1:400) and rabbit anti-laminin (L9393 - Sigma; diluted 1:50). Secondary FITC-conjugated antibody goat anti-rabbit (F9259 - Sigma) was also used in the reaction. All incubations were performed for 40 min with antibodies diluted in PGN-saponin. After the washes in PBS, the slides were mounted in glycerol containing 0.1% p-phenylenediamine (Sigma). The slides were examined in a confocal scanning microscope LSM 510-META (Zeiss).

### Laminin and fibronectin quantification

Six images of each tissue section were obtained by confocal laser scanning microscopy. The software Image Pro Plus 4.0 was used to measure the area, in μm^2^, of the laminin and fibronectin multifunctional proteins. The mean of measured areas was calculated and afterwards compared among studied mice strains.

### Statistical analysis

The Kruskal-Wallis test was used for statistical analysis of the laminin and fibronectin quantification results.

## Results

### Kinetic of lesions

**Figure 1:**
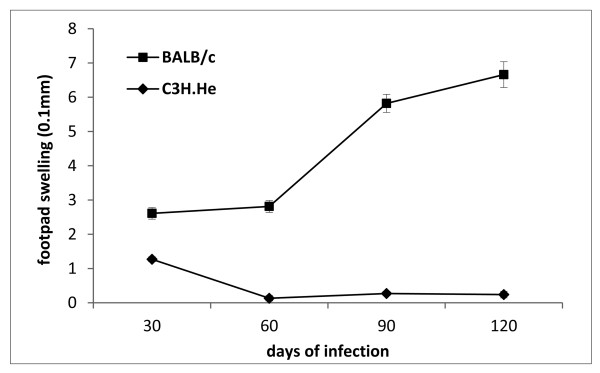
**Footpad swelling in BALB/c and C3H.He mouse strains infected by 10^4 ^*L*. *amazonensis *amastigotes (H21 MHOM/BR/76/MA-76) in the left hind footpad**. Each point represents the average increase in footpad thickness ± SEM (*n *= 5).

Figure 1 shows that in the initial phase, both mice strains, presented no differences in the size of the footpad, but along the course of the infection, the lesion increased in BALB/c mice.

### Histopathology analysis

**Figure 2:**
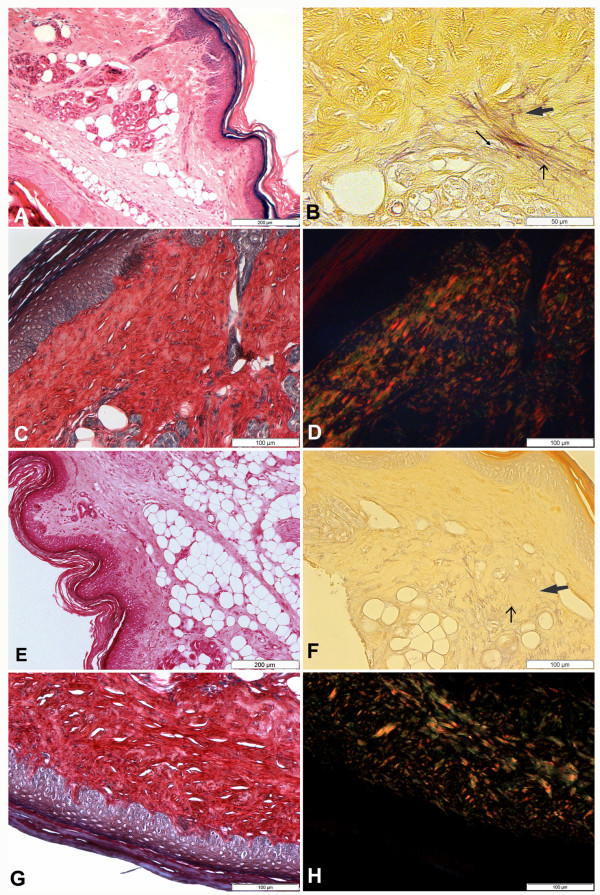
**Histopathological analysis of skin fragments from left footpad of BALB/c (A, B, C and D) and C3H.He (E, F, G and H) mock-infected mice, 30 days after mock infection**. **A**. Normal morphology of footpad is observed. The integrity of the epidermis and dermis, the presence of adipocytes and ducts of regular glands can be seen (HE). **B**. Elastic (thin arrow), elaunin (thick arrow) and oxytalan (arrow) fibres with normal morphology and location are also observed (Weigert). **C**: Picrosirius red in non-polarized light showing the regular arrangement of the skin showing epidermis, dermis and hypodermis. **D**. Picrosirius red in polarized light shows the normal arrangement of type I collagen (orange and yellow) and type III (green). **E**. Normal morphology of footpad is observed. The organization of the epidermis and dermis were maintained, the presence of adipocytes and ducts of regular glands can also be seen (HE). **F**. Elastic fibres (thin arrow), elaunin fibres (thick arrow) with normal morphology and location are noted (Weigert). **G**. Picrosirius red in non-polarized light showing the regular organization of the skin showing epidermis, dermis and hypodermis. **H**. Picrosirius red polarized light shows the normal organization of type I collagen (orange and yellow) and type III (green).

BALB/c mock-infected mice footpad analysis showed normal morphology of tissues under light microscopy. Skin layers, epidermis, dermis and hypodermis, were well preserved. Internal layers could also be seen, especially the subcutaneous adipose tissue (Figure 2a). Elastic, elaunin and oxytalan fibres were demonstrated by Weigert's staining pre-oxidation (Figure 2b). Type I collagen and type III was noted (Figure 2c and 2d). C3H.He mice mock-infected also showed, as observed in BALB/c mice, well preserved footpad tissues. Figure 2e shows the epidermis, dermis and hypodermis and subcutaneous adipose tissues.

Figure 2f shows the elastic fibre systems: the elastic fibres and also the elaunin fibres, which are less intensively stained than the elastic fibres. Oxytalan fibres were not seen in C3H.He mice. The distribution of collagen type I and type III, stained with picrosirius red in non-polarized (Figure 2g) and polarized light (Figure 2h) was also normal in this mouse line.

**Figure 3:**
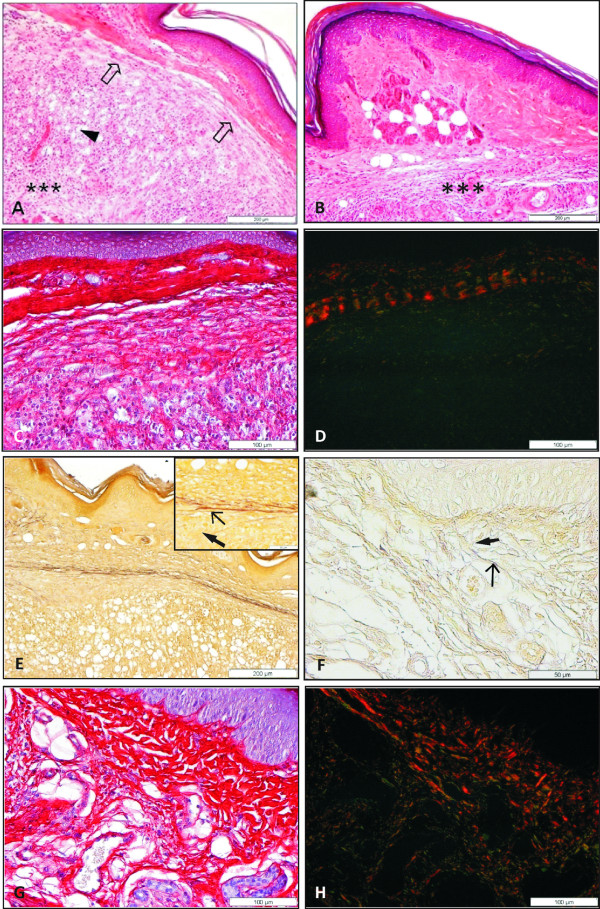
**Histopathological analysis of footpad skin from mice subcutaneously infected with 10^4 ^amastigotes of *L. amazonensis***. **A**. BALB/c mice 30 days after infection. Intense inflammatory infiltrate in the reticular dermis (***), with numerous vacuolated macrophages (arrowheads) and polymorphonuclear cells, and slight dissociation of the extracellular matrix fibres (plain arrow) (HE). **B**. C3H.He mice 30 dpi. Intact papillar dermis and moderate inflammatory infiltrate (***) rich in neutrophils and parasitized macrophages in the reticular dermis (HE). **C **and **D**: BALB/c mice 30 days after infection. Picrosirius red in non-polarized (C) and polarized light (D). The image shows type I collagen (red) and a significant production of collagen type III (green) between inflammatory cells present in the reticular dermis. **E**. BALB/c mice 30 dpi. Window shows elastic (thin arrow) and elaunin fibres (thick arrow) preserved. **F**. C3H.He mice 60 days after infection. Integrity of elastic (thin arrow) and elaunin (thick arrow) and absence of oxytalan fibres (Weigert). **G **and **H**: C3H.He mice 60 days after infection. Picrosirius red in non-polarized (G) and polarized light (H). Attempt to repair infected dermis, with presence of collagens type I (red), III and I neoformed (yellow).

Thirty days after infection an intense inflammatory infiltrate in the reticular dermis, with polymorphonuclear (PMN) cells and vacuolated macrophages full of parasites, was observed in BALB/c and C3H.He mice. Infected BALB/c mice presented changes in the matrix of reticular dermis which is characterized by degradation of type I collagens among inflammatory cells (Figure 3a). In BALB/c mice, picrosirius red stain showed a significant production of type III collagen sustaining inflammatory cells in reticular dermis (Figure 3c, d). While in papillary dermis only a discreet inflammatory process was observed and no alterations of type I and III collagens were noted in infected BALB/c or in control mice. In the papillary dermis of infected C3H.He mice, where the inflammatory reaction was discreet or absent (Figure 3b), the distribution of type I and III collagens was similar to control group.

In both mouse strains the elastic system fibres showed preservation of elastic and elaunin fibres, however oxytalan fibres were not detected in areas associated to the inflammatory process (Figure 2e). Elastic fibres, near the parasitized cells, were not degraded but presented a different pattern of distribution when compared with control mice.

### 60 dpi

The BALB/c mice presented densely parasitized dermis, extensive areas of necrosis and a reduction of collagen fibres. The elastic and elaunin fibres did not undergo changes; however type I collagen presented a remarkable reduction when compared with control mice.

The C3H.He mice showed an intense inflammatory infiltrate composed basically of PMN and a few parasitized macrophages. The picrosirius red revealed that these mice, when compared with BALB/c, presented a moderate alteration of their ECM components. Also in C3H.He mice there was an enhancement of type III collagen, confirmed by Gomori reticulin stain, associated to a large number of inflammatory cells. A neoformation of type I and III collagen was noted in some C3H.He mice dermis (Figure 3g, h). The integrity of the elastic and elaunin fibres was maintained the oxytalan fibres were not observed (Figure 3f).

### 90 dpi

In BALB/c mice an intense inflammatory infiltrate and an enhancement of densely parasitized macrophages was observed. At this time of infection, due to tissual damage, type III collagen associated to inflammatory cells was no longer observed. The elastic and elaunin fibres were preserved at the dermal-epidermal junction.

**Figure 4:**
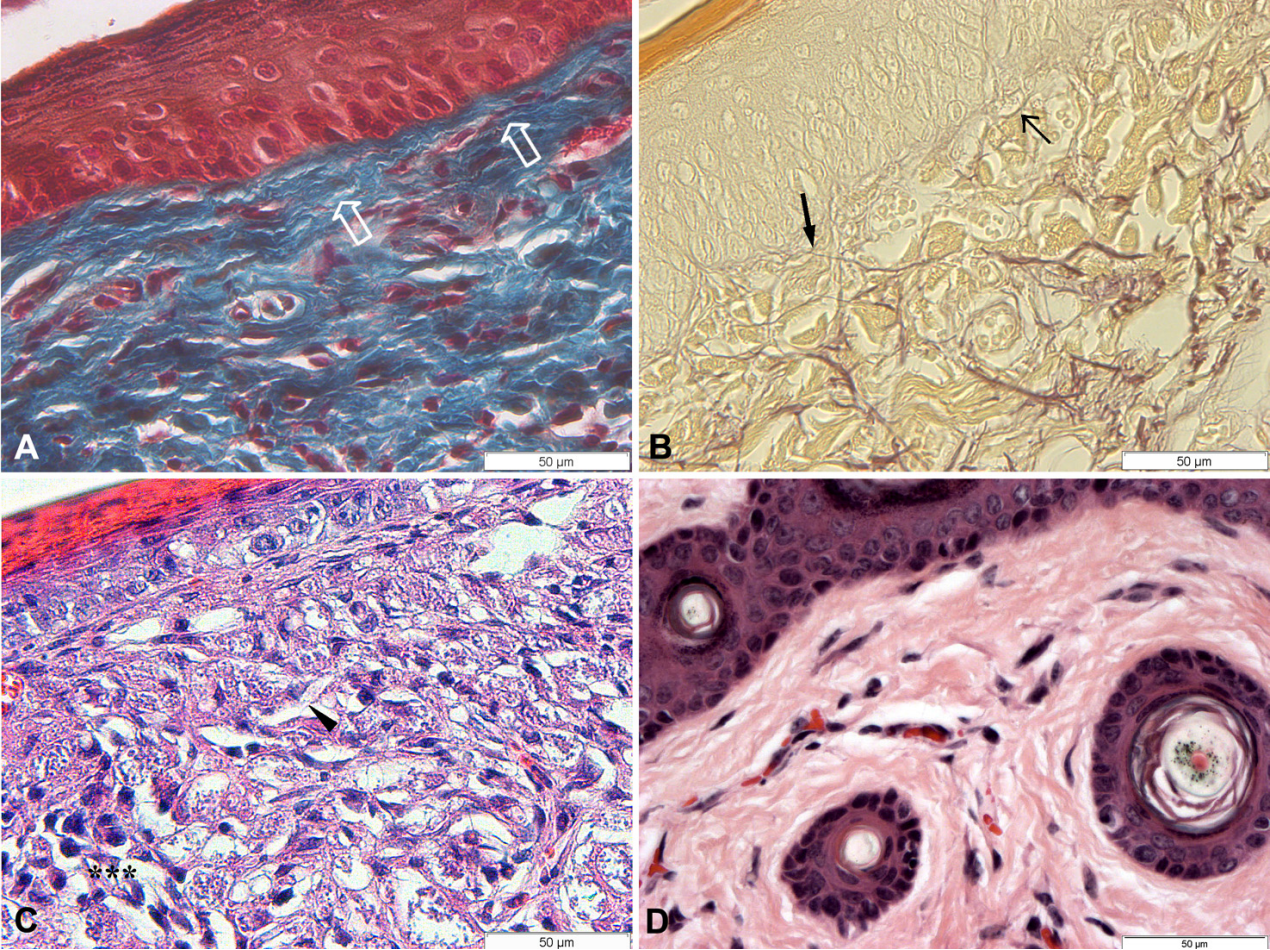
**Histopathological analysis of footpad skin from mice subcutaneously infected with 10^4 ^amastigotes of *L. amazonensis***. **A **and **B**: C3H.He mice 90 days after infection. **A**: Proliferation and arrangement of collagen fibre (plain arrow) compatible to dermis restoration (Masson); **B**: Dermo-epidermic junction shows preserved elastic (thin arrow) and elaunin fibres (thick arrow) (Weigert); **C**: BALB/c mice 120 days after infection. Diffuse inflammatory infiltrate (***) composed of PMN and macrophages heavily parasitized (arrowhead); **D**: C3H.He mice 120 dpi. (HE). It was observed an absence of lesions and regeneration of tissue architecture.

The C3H.He mice showed a discreet inflammatory infiltrate, absence of parasitized cells, and discreet disaggregation of fibres from ECM. The distribution of type I and III collagen was compatible to a reparation process. Masson's trichrome stain showed proliferation and rearrangement of collagen fibres confirming the process of dermal reparation (Figure 4a). The elastic and elaunin fibres of the dermal-epidermal junction were preserved (Figure 4b).

### 120 dpi

At the infection site of BALB/c mice there was a diffused inflammatory infiltrate, constituted by PMN cells and intensively parasitized macrophages, as well as free parasites, degraded collagen fibres, and destruction of the epidermal layers (Figure 4c), furthering the contact between parasites and keratinocytes. Also degradation of type I and III collagen in the dermal-epidermal junction was seen. There was, in addition, a degradation of type I and III collagen fibres in the papillary dermis at 120 dpi.

C3H.He mice presented neither clinical nor histopathological alterations. The histological findings were similar to those observed in non-infected mice (Figure 4d), including the same pattern of ECM distribution. Also the elastic fibres were preserved.

### Confocal analysis

**Figure 5:**
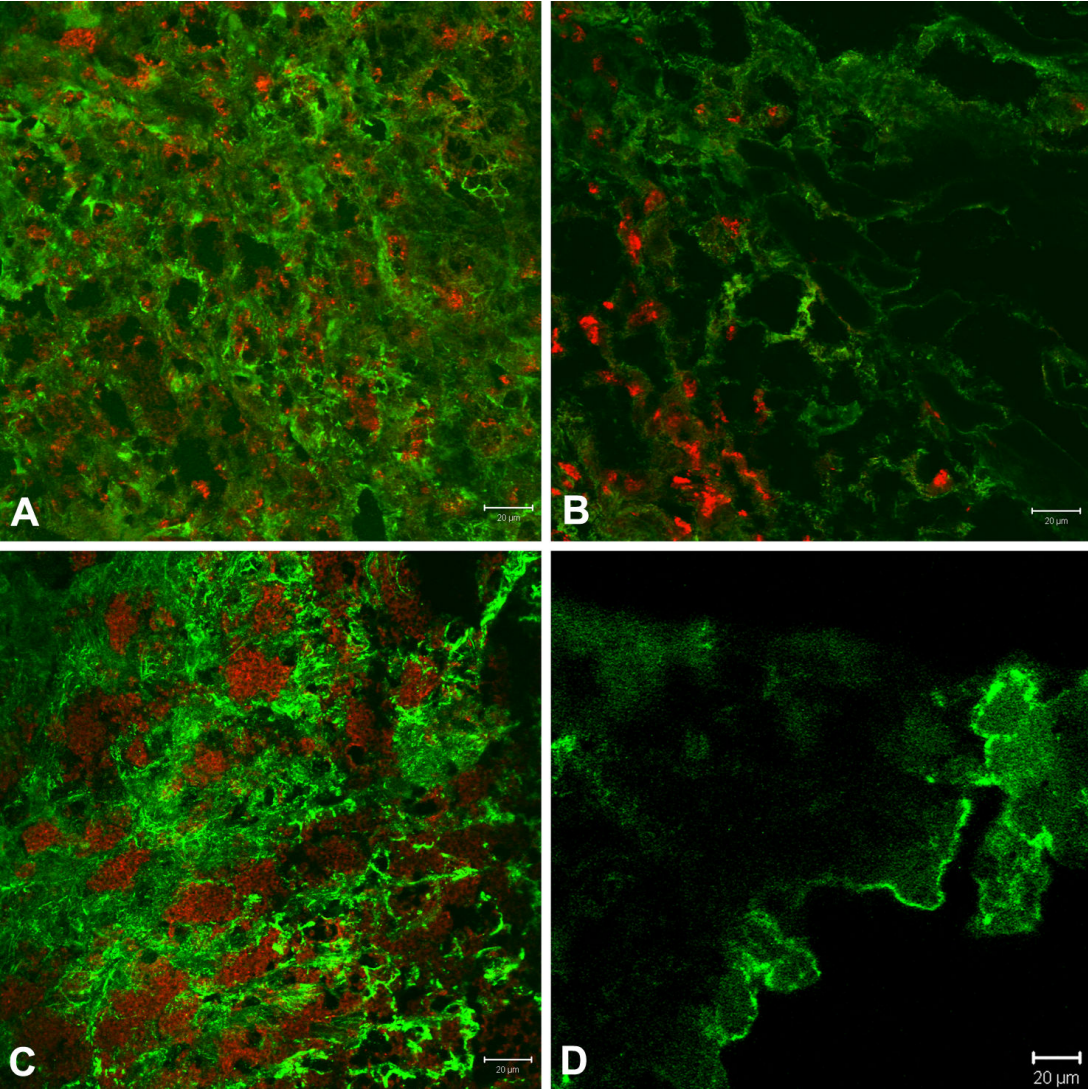
**Immunohistochemical analysis of skin fragments from the left hind footpad from mice subcutaneously infected with 10^4 ^*L. amazonensis *amastigotes**. Fibronectin is labeled in green (FITC) and *L. amazonensis *amastigotes in red (Cy3): **A: **BALB/c infected mice and **B: **C3H.He infected mice 30 days after infection; **C: **BALB/c infected mice and **D: **C3H.He infected mice 120 days after infection.

Fibronectin and laminin confocal analysis, thirty days after infection, showed that infected BALB/c mice presented an enhancement of fibronectin when compared with non-infected control (*p *< 0.001) (Figure 5a) and with infected C3H.He mice (*p *< 0.05) (Figure 5b). However, when mice were evaluated on the 120th day of infection, infected C3H.He mice presented a reduction of this protein when compared with infected BALB/c mice (*p *< 0.001) or with non-infected controls (Figure 5c, d). When laminin was analyzed no significant alterations were observed in the two mice strains studied.

## Discussion

When *Leishmania *sp. parasites are regurgitated by the vector as promastigotes or when released by macrophage lysis as amastigotes into the skin [11] they are in contact with the extracellular microenvironment and, consequently, interact with the ECM constituents [6].

In the present study we observed alterations in the collagenous components of ECM in BALB/c mice, caused by *Leishmania *infection. There was a significant synthesis of type III collagen of this mouse strain in the first months of infection, mainly where the inflammatory process was more intense. This enhancement is due to the capacity of type III collagen to confer sustentation to the inflammatory cells that were in large numbers at the infection site at this time. As the infection progressed the number of parasitized macrophages increased substantially, occupying the space of the ECM constituents. So, in the advanced stages of the infection neither type III nor type I collagen was observed. The latter had already been degraded by the 60^th ^day after infection in most of the BALB/c mice. Abreu-Silva et al. [12] described the same collagen replacement during *L. amazonensis *infection in BALB/c mice. An in vitro study, with the promastigote stage, demonstrated the ability of *L*. *mexicana *to bind to type I collagen, indicating that this interaction could be important in the pathogenesis of the infection at the onset of specific parasite tropism for host skin [8].

C3H.He mice also exhibited an enhancement of type III collagen in the initial days of infection, when there was a larger amount of inflammatory cells. However, after 60 dpi the resurgence of type I collagen was noticeable in some mice of this strain. This is consistent with the attempt to maintain tissue architecture and to restore the dermis, without fibrosis formation, which characterizes a repair by scarring.

The role played by the elastic system in parasitic diseases has not been extensively studied, including in leishmaniasis. In this disease there are few reports concerning the alterations of these fibres [13]. Our data shows that the oxytalan fibres are absent from the inoculation site 30 dpi, while elastic and elaunin fibres, insoluble fibres due to presence of elastin [4,14], are preserved in both mice strains. However, as of the 120^th ^day of infection in the C3H.He mouse strain, the oxytalan fibres had returned in the composition of the ECM tissue. This repair is probably related to the genetic background of this mouse [15].

According to Ghosh et al. [6,16] there is a laminin binding protein on the surface of promastigotes of *L*. *donovani *that may mediate the cell adhesion. The ability of this protein to bind to ECM proteins such as laminin probably influences the clinical course of the disease that this species causes in their hosts. This ability may allow parasite persistence in the host contributing to its pathogenicity. Besides, it can also facilitate the parasite adhesion to host cells such as macrophages, via laminin receptors present on the cell surface [9]. Subsequently, El Ghalbzouri et al. [17] demonstrated in vitro, the expression of laminin by keratinocytes and reported that this protein mediates the binding of these cells to the dermal compartment only when fibroblasts or exogenous growth factors are present. Thus, these authors demonstrated the importance of the interaction between epithelial and mesenchymal cells in the regulation of basement membrane component synthesis.

The dermis comprises two layers without distinct boundaries: (1) the papillary layer, consisting of loose connective tissue (fibroblasts, collagen fibres and thin elastic) in close contact with the epidermis; and (2) the reticular layer, containing thick bundles of collagen fibres and coarse elastic fibres. The skin represents a barrier to parasite mobility and its access to nutrients [18]. The *L*. *amazonensis *is preferably dermotropic and has as a characteristic a strong involvement with the ECM components, collagens in general and especially with multifunctional proteins such as fibronectin and laminin [12,19]. So, we can say that the presence of the parasite influences the synthesis of fibronectin and laminin which in turn interferes with the adherence, endocytosis and subsequent internalization of the parasites. Silva [20] demonstrated, in vitro, a relationship between the profile of fibronectin and laminin production and the internalization of *L*. *amazonensis *promastigotes in retinal pigmented epithelial cells. The increase of the expression of these proteins was directly associated with a high number of parasites adhered and internalized in these cells.

In the present study we observed an enhancement of fibronectin in BALB/c mice, susceptible to *L*. *amazonensis *that had more serious lesions, with the presence of many free parasites or within macrophages, associated with a severe inflammatory infiltrate. In C3H.He mice, less susceptible to infection, the lesions were smaller, with fewer parasites and fibronectin was present in smaller amounts. These results were similar to those described by Calvet et al. [21] in in vivo *Trypanosoma cruzi *experimental infection, who observed an enhancement of fibronectin associated with the inflammatory infiltrate in Swiss mice.

The evaluation of laminin showed no significant difference in this protein production in C3H.He or BALB/c infected or control mice, corroborating the findings of Abreu-Silva et al. [12]. According to Kulkarni et al. [22] the degradation of laminin by *Leishmania *may contribute to the disruption of the ECM, facilitating the local spread of the parasite.

Our study evaluated the histopathological alterations in the ECM compounds in the *L. amazonensis *infection. Most of the studies related to this subject have been done in vitro using promastigotes. Our group was the first to analyze these alterations in vivo [12], and with the present study we can conclude that C3H.He mice exhibit the capacity to restore the ECM compounds by the 120^th ^day of infection, demonstrating a relative resistance against *L. amazonensis *while there was an intense destruction of the ECM during the infection of BALB/c mice.

## Competing interests

The authors declare that they have no competing interests.

## Authors' contributions

KSC and LOPC participated in the design of the study and made the final interpretation of the data. MSA, CSFS and DJH carried out mice inoculation, necropsies and kinetic of lesions. LOPC and DJH prepared the histology. LOPC and MSA performed histopathological diagnosis. MSA and DJH prepared the immunohistochemistry, laminin and fibronectin quantification and statistical analysis and participated in interpretation of data. MSA, LOPC, ALAS and KSC prepared the manuscript. All authors read and approved the final manuscript.
